# EspalomaCharge: Machine Learning-Enabled Ultrafast Partial Charge Assignment

**DOI:** 10.1021/acs.jpca.4c01287

**Published:** 2024-05-08

**Authors:** Yuanqing Wang, Iván Pulido, Kenichiro Takaba, Benjamin Kaminow, Jenke Scheen, Lily Wang, John D. Chodera

**Affiliations:** † Computational and Systems Biology Program, Sloan Kettering Institute, Memorial Sloan Kettering Cancer Center, New York, New York 10065, United States; ‡ Simons Center for Computational Chemistry and Center for Data Science, New York University, New York, New York 10004, United States; § Pharmaceutical Research Center, Advanced Drug Discovery, Asahi Kasei Pharma Corporation, Shizuoka 410-2321, Japan; ∥ Tri-Institutional PhD Program in Computational Biology and Medicine, Weill Cornell Medical College, Cornell University, New York, New York 10065, United States; ⊥ Open Molecular Sciences Foundation, Davis, California 95618, United States

## Abstract

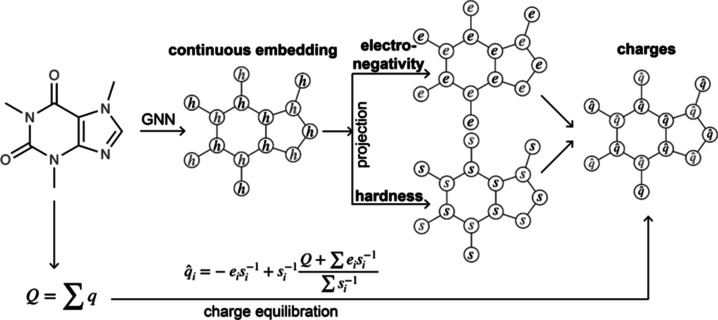

Atomic partial charges are crucial parameters in molecular dynamics simulation, dictating the electrostatic contributions to intermolecular energies and thereby the potential energy landscape. Traditionally, the assignment of partial charges has relied on surrogates of ab initio semiempirical quantum chemical methods such as AM1-BCC and is expensive for large systems or large numbers of molecules. We propose a hybrid physical/graph neural network-based approximation to the widely popular AM1-BCC charge model that is orders of magnitude faster while maintaining accuracy comparable to differences in AM1-BCC implementations. Our hybrid approach couples a graph neural network to a streamlined charge equilibration approach in order to predict molecule-specific atomic electronegativity and hardness parameters, followed by analytical determination of optimal charge-equilibrated parameters that preserve total molecular charge. This hybrid approach scales linearly with the number of atoms, enabling for the first time the use of fully consistent charge models for small molecules and biopolymers for the construction of next-generation self-consistent biomolecular force fields. Implemented in the free and open source package EspalomaCharge, this approach provides drop-in replacements for both AmberTools antechamber and the Open Force Field Toolkit charging workflows, in addition to stand-alone charge generation interfaces. Source code is available at https://github.com/choderalab/espaloma-charge.

## Introduction

Molecular mechanics (MM) force fields abstract atoms as point charge-carrying particles, with their electrostatic energy (*U*_e_) calculated by some Coulomb’s law^6^
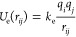
1(or some modified form), where *k*_e_ is Coulomb constant (energy * distance^2^/charge^2^) and *r*_*ij*_ the interatomic distance. In fixed-charge MMs force fields, the partial charges *q*_*i*_ are treated as constant, static parameters, independent of instantaneous geometry. As such, partial charge assignment—the manner in which partial charges are assigned to each atom in a given system based on their chemical environments—plays a crucial role in molecular dynamics (MD) simulation, determining the electrostatic energy (*U*_e_) at every step and shaping the energy landscape.

### Traditionally, Partial Charges Have Been Derived from Expensive Ab Initio or Semiempirical Quantum Chemical Approaches

In the early stages of development of molecule mechanics (MM) force fields, ab initio methods were used to generate electrostatic potentials (ESP) on molecular surfaces from which restrained ESP (RESP) charge fits were derived.^2^ This process proved to be expensive, especially for large molecules or large numbers of molecules (e.g., in virtual screening, where data sets now approach 10^9^ molecules^11^). This led to the development of the AM1-bond charge correction (BCC) charge scheme^16,17^—a method for approximating RESP fits at the HF/6-31G* level of theory, by first calculating population charges using the much less expensive AM1 semiempirical level of theory and subsequently correcting charges via BCCs. As a result, this approach has been widely adopted by the MMs community utilizing force fields such as GAFF^28^ and the open force fields.^26^

Despite this progress, there are still multiple drawbacks with AM1-BCC. First, the computation is dependent on the generation of one or more conformers, which contributes to the discrepancy among the results of different chemoinformatics toolkits. While conformer ensemble selection methods such as ELF10^a^ attempt to minimize these geometry-dependent effects, they do not fully eliminate them, and significant discrepancies between toolkits can remain.

Second, the speed is still a bottleneck (especially when it comes to the virtual screening of large libraries) as it still requires QM calculation for the parametrization. Moreover, the runtime complexity of AM1-BCC scales  in the number of atoms *N*. In particular, the poor runtime complexity necessitates using a different charging model for biopolymers (such as proteins and nucleic acids), making the process of extending these polymeric force fields to accommodate post-translational modifications, nonstandard residues, covalent ligands, and other chemical modifications both complex and likely to require a third charging strategy within the same simulation.

### Machine Learning Approaches to Charge Assignment Have Recently Been Proposed but Face Challenges in Balancing Generalization with the Ability to Preserve Total Molecular Charge

The rising popularity of machine learning has led to a desire to exploit new approaches to rapidly predict partial atomic charges. For example, recent work from Bleiziffer et al.^4^ employed a random forest approach to assign charges based on atomic features but faced the issue of needing to preserve total molecular charge while making predictions on an atomic basis—they distribute the difference between predicted and reference charge evenly among atoms. Similarly, Metcalf et al.^22^ preserve the total charge by allowing only charge transfer in message-passing form resulting in zero net-charge change. A more classical approach by Gilson et al.^10^ tackles the charge constraint problem in a clever manner: instead of directly predicting charges, by predicting atomic electronegativity and electronic hardness, a simple constrained optimization problem inspired by physical charge equilibration (QEq)^27^ can be solved analytically to yield partial charges that satisfy total molecular charge constraints. In spite of its experimental success, its ability to reproduce quantum-chemistry-based charges is heavily dependent upon the discrete atom typing scheme to classify and group atoms by their chemical environments. Additionally, charges have been considered in new deep machine learning potential models,^20^ and machine learning has also been employed to come up with electrostatic parameters for Drude oscillator force fields.^21^

**Figure 1:**
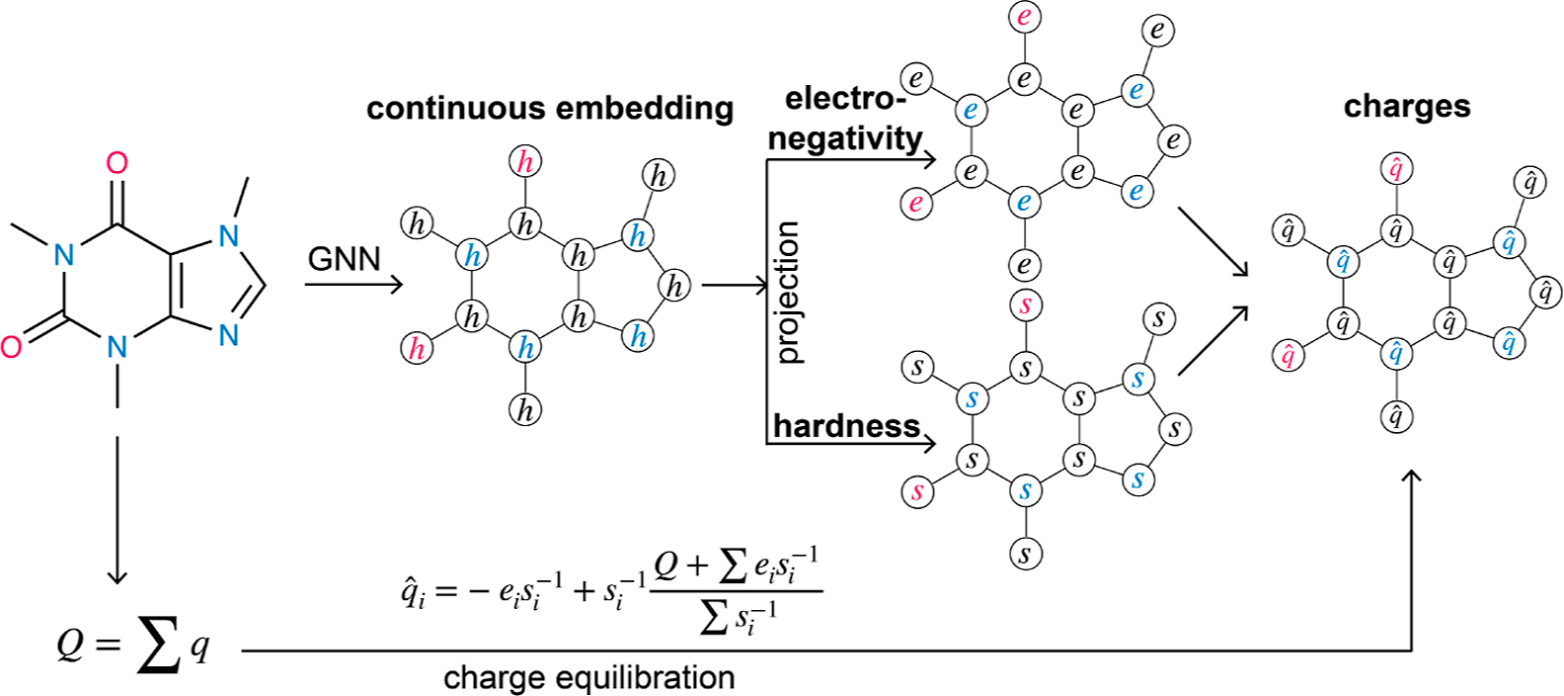
Schematic overview of EspalomaCharge: a hybrid physical/GNN model for fast charge assignment. First, the graph node representation *h* assigned by a GNN is used to compute unconstrained electronegativity *e*_*i*_ and hardness *s*_*i*_ to each atom. Second, the charge potential energy is minimized analytically to yield predicted partial charges  that satisfy the total molecular charge constraint *Q*.

Recently, Wang^29^ and Wang et al.^31^ designed a graph neural networks-based atom typing scheme, termed **Espaloma** (extensible surrogate potential optimized by message-passing algorithms), to replace the human expert-derived, discrete atom types with continuous atom embeddings (Figure 1). This allows atoms with subtle chemical environment differences to be distinguished by the model without the need to painstakingly specify heuristics.
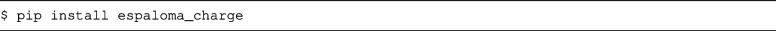

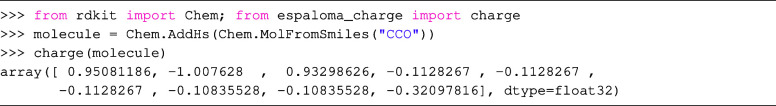


### EspalomaCharge Generates AM1-BCC ELF10 Quality Charges in an Ultrafast Manner Using Machine Learning

In this paper, we use the continuous embedding atom representation scheme from **Espaloma** in conjunction with analytical constrained charge assignment inspired by QEq to come up with an ultrafast machine learning surrogate for partial charge assignment (EspalomaCharge). We train EspalomaCharge on an expanded set of protonation states and tautomers of representative biomolecules and drug-like molecules (the SPICE data set^8^) to assign high-quality AM1-BCC ELF10 charges.^17^ The resulting EspalomaCharge model accurately reproduces AM1-BCC ELF10 charges to an error well within the discrepancy between AmberTools sqm and OpenEye oequacpac implementations on average 2000 times faster than AmberTools on the SPICE data set, can utilize either CPU or GPU, and scales as  with a number of atoms, allowing even entire proteins to be assigned AM1-BCC equivalent charges. We implement this approach in the Python package EspalomaCharge, which is distributed open source under an MIT license and pip-installable (Listing 1).

## Theory: Espaloma Graph Neural Networks for Chemical Environment Perception, QEq, and EspalomaCharge

### Espaloma Uses Graph Neural Networks to Perceive Atomic Chemical Environments

**Espaloma**(31) uses graph neural networks (GNNs)^1,9,14,19,30,34^ to assign continuous latent representations of chemical environments to atoms that replace human expert-derived discrete atom types. These continuous atom representations are subsequently used to assign symmetry-preserving parameters for atomic, bond, angle, torsion, and improper force terms.

When GNNs are employed in chemical modeling, the atoms are abstracted as nodes (*v*) and bonds as edges (*e*) of a graph . *h*_v_^(0)^, the initial features associated with node *v* are determined based on resonance-independent atomic chemical features from a cheminformatics toolkit (see Supporting Information section). Following the framework from Battaglia et al.,^1^ Gilmer et al.,^9^ and Xu et al.,^34^ for a node *v* with neighbors , in a graph , with *h*_v_^(*k*)^ denoting the feature of node *v* at the *k*-th layer (or *k*-th round of message-passing) and  the initial node feature on the embedding space, the *k*-th message-passing step of a GNN can be written as three steps: first, an edge update
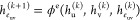
2where the feature embeddings *h*_u_ of two connected nodes u and v update their edge feature embedding , followed by neighborhood aggregation
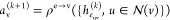
3where edges incident to a node *v* pool their embeddings to form aggregated neighbor embedding *a*_v_, and finally, a node update
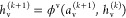
4where  denotes the operation to return the multiset of neighbors of a node and ϕ^e^ and ϕ^v^ are implemented as feed-forward neural networks. Since the neighborhood aggregation functions ρ^e→v^ are always chosen to be indexing-invariant functions, namely, SUM or MEAN operator, eq 3, and thereby the entire scheme, is permutationally invariant. In practice, choices such as the dimensionality of node and edge vectors, number of layers, layer width, activation function, aggregation operators, and initial conditions for training are treated as hyperparameters and optimized during training to produce robust, near-optimal models on a held-out validation set separate from a test set.

### QEq Is a Physically Inspired Model for Computing Partial Charges while Maintaining Total Molecular Charge

This **Espaloma** framework can be used to predict atomic parameters that can be fed into subsequent neural modules that predict MMs parameters. For partial charges, however, the constraint that the predicted partial charges  should sum up to the total charge *Q*—the sum of all formal charges or total molecular charge—is nontrivial to satisfy were the charges to be predicted directly.
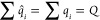
5We adopt the method proposed by Gilson et al.^10^ where we predict the electronegativity *e*_*i*_ and hardness *s*_*i*_ of each atom *i*, which are defined as the first- and second-order derivative of the potential energy in QEq approaches^27^
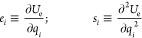
6Next, we minimize the second-order Taylor expansion of the charging potential energy contributed by these terms, neglecting interatomic electrostatic interactions
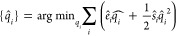
7which, as it turns out, has an analytical solution given by Lagrange multipliers
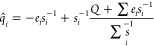
8We thus use the Espaloma framework to predict the unconstrained atomic electronegativity (*e*) and hardness (*s*) parameters used in eq 8 to assign partial charges in a manner that ensures that total molecular charge sums to *Q*. It is worth noting that, by the equivalence analysis proposed in Wang et al.,^31^ the tabulated atom typing scheme Gilson et al.^10^ uses amounts to a model working analogously to a Weisfeiler-Lehman test^33^ with hand-written kernel, whereas here we replace this with an end-to-end differentiable GNN model to greatly expand its resolution and ability to optimize based on reference charges.

### EspalomaCharge Has  Time Complexity in the Number of Atoms

One of the primary advantages of spatial GNNs that pass messages among local neighborhoods is their  complexity, where *E* is the number of edges. In chemical modeling, since the sparsity of the graph is roughly fixed (the number of edges is 3 to 4 times that of number of nodes), it is safe to write the runtime complexity as , with *N* being the number of nodes (atoms). The QEq step with its linear operator does not alter the complexity nor is it the bottleneck of EspalomaCharge. Therefore, unlike with ab initio or semiempirical methods, the runtime complexity of EspalomaCharge is .

## Experiments: EspalomaCharge Accurately Reproduces AM1-BCC Charges at a Fraction of Its Cost

We show, in this section, that the discrepancy between EspalomaCharge and the OpenEye toolkit is comparable to or smaller than that between AmberTools^5^ and OpenEye. EspalomaCharge is fast and scalable to larger systems, taking seconds to parameterize a biopolymer with 100 residues on CPU.

### SPICE Data Set Covers Biochemically and Biophysically Interesting Chemical Space

To curate a data set representing the chemical space of interest for biophysical modeling of biomolecules and drug-like small molecules, we use the SPICE^8^ data set, enumerating reasonable protonation and tautomeric states with the OpenEye Toolkit. We generated AM1-BCC ELF10 charges for each of these molecules using the OpenEye Toolkit and trained EspalomaCharge (Figure 1) to reproduce the partial atomic charges with a squared loss function. This model, with its parameters distributed with the code, is used in all of the characterization results hereafter.

### EspalomaCharge Is Accurate, Especially on Chemical Spaces Where Training Data Is Abundant

First, upon training on the 80% training set of SPICE, we test on the 10% held-out test set to benchmark the in-distribution (similar chemical species) performance of EspalomaCharge (Table 1, first half). Notably, the discrepancy [measured by charge root-mean-square error (RMSE)] between EspalomaCharge and OpenEye is comparable with or smaller than that between AmberTools^5^ and OpenEye—two popular chemoinformatics toolkits for assigning AM1-BCC charges to small molecules. Since it is a common practice in the community to use these two toolkits essentially interchangeably, we argue that the discrepancy between these could be established as a baseline below which the error is no longer meaningful.

**Table 1:** EspalomaCharge Accurately and Efficiently Reproduces AM1-BCC Charges for a Wide Variety of Chemical Spaces^a^

			average RMSE (e)				average walltime (s)		
data set	*N*_mol_	avg. *N*_atoms_	|EspalomaCharge–OpenEye|		|AmberTools–OpenEye|		EspalomaCharge	AmberTools	OpenEye
SPICE^8^ test set	29079	39.36	**0.0435**	0.0438	0.0623	0.0628	**0.05**	93.10	3.79
				0.0432		0.0618			
FDA approved	1019	34.80	**0.0266**	0.0255	**0.0244**	0.0263	**0.03**	46.15	1.87
				0.0281		0.0227			
ZINC250 K^12^	220250	42.70	**0.0187**	0.0187	0.0197	0.0198	**0.05**	124.89	3.63
				0.0187		0.0197			
FreeSolv^7^	641	18.10	0.0110	0.0117	**0.0067**	0.0077	**0.03**	9.62	0.43
				0.0104		0.0057			
PDB expo^3^	23399	35.94	**0.0186**	0.0188	0.0232	0.0236	**0.04**	88.86	3.63
				0.0184		0.0229			

a Here, *N*_mol_ denotes the number of molecules in the data set; avg. *N*_atoms_ denote the average number of atoms in molecules for the corresponding data set; average RMSE is the charge RMS deviation between AM1-BCC implementations averaged over all molecules in the data set, with sub- and superscripts denoting the 95%-confidence interval of the mean (computed by bootstrapping over molecules in the data set with replacement); average wall time denotes the average wall time for the respective toolkit to assign partial charges for a molecule in the data set. Boldface statistics denote the best (most accurate or fastest) model or models (in case confidence intervals are indistinguishable) for each statistic.

We prepare several out-of-distribution external data sets to test the generalizability of EspalomaCharge to other molecules of significance to chemical and biophysical modeling, including a filtered list of FDA-approved drugs, a subset of the ZINC^12,15^ purchasable chemical space, and finally the FreeSolv^23^ data set consisting of molecules with experimental and computationally estimated solvation free energy. The discrepancy between EspalomaCharge and OpenEye is lower than or comparable with that between AmberTools and OpenEye, demonstrating that the high performance of EspalomaCharge is generalizable, at least within chemical spaces frequently used in chemical modeling and drug discovery.

**Figure 2:**
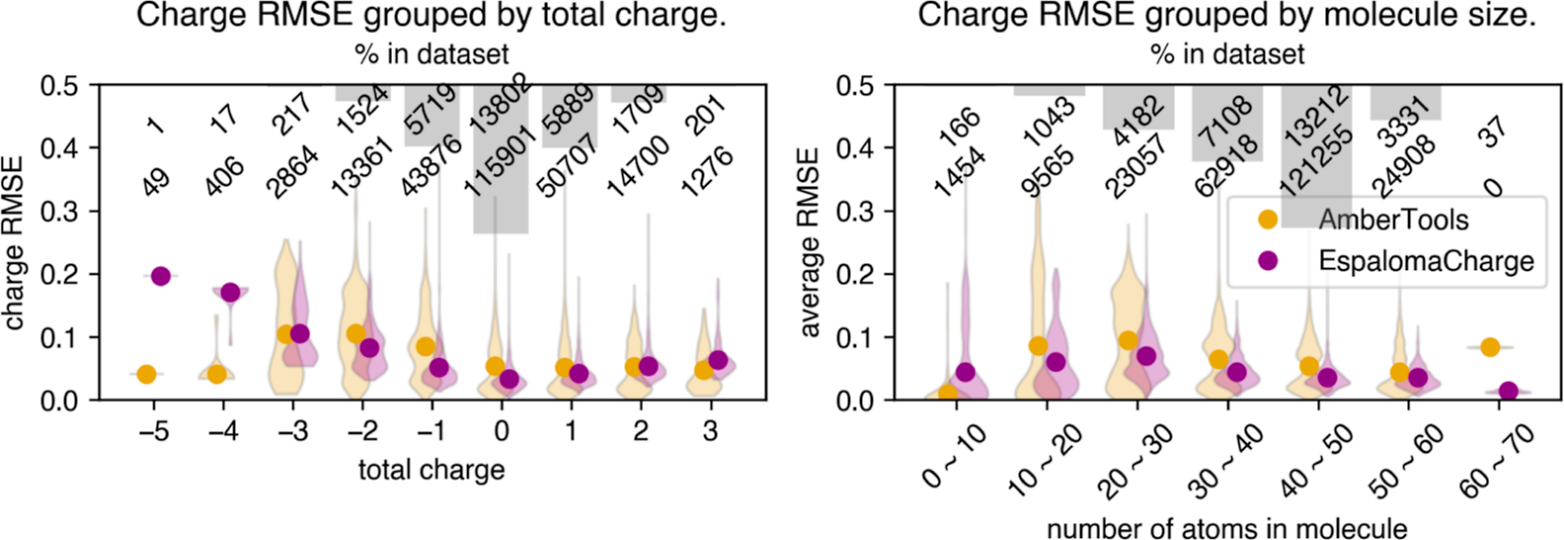
EspalomaCharge shows smaller average charge RMSE than AmberTools on well-represented regions of chemical space. SPICE data set test set performance stratified by total charge (left panel) and molecule size (right panel). To better illustrate the effects of limited training data on stratified performance, the number of test (upper number) and training (lower number) molecules falling into respective categories are also annotated with test set distribution plotted as histogram.

To pinpoint the source of the error for EspalomaCharge, we stratified the molecules by the number of atoms and total molecular charge, computing the errors on each subset (Figure 2). Compared to the error baseline, EspalomaCharge is most accurate where there was abundant data in the training set. This is especially true when it comes to stratification by net molecular charge since the extrapolation from small systems to larger systems is encoded in the inductive biases of GNNs. Given the performance of well-sampled charge bins, it seems likely the poor performance for molecules with more exotic −4 and −5 net charges will be resolved once the data set is enriched with more examples of these states.
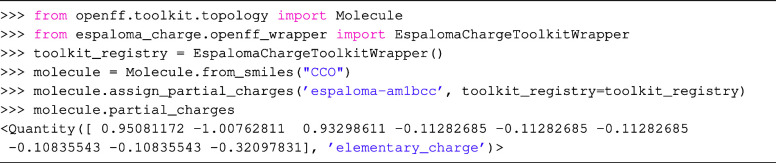

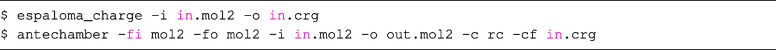


It is worth mentioning that unified application programming interfaces (API) (Listing 3) integrated in Open Force Field toolkits are responsible for generating the performance benchmark experiments above. Additionally, a command–line interface (CLI) is also provided for seamless integration of EspalomaCharge into Amber workflows (Listing 4).

### EspalomaCharge Is Fast, Even on Large Biomolecular Systems

Apart from the accurate performance, the drastic difference in the speed of parametrization is also observed in the benchmarking experiments. For the small molecule data sets in Table 1, EspalomaCharge is 300–3000 times faster than AmberTools and 15–75 times faster than OpenEye.

**Figure 3:**
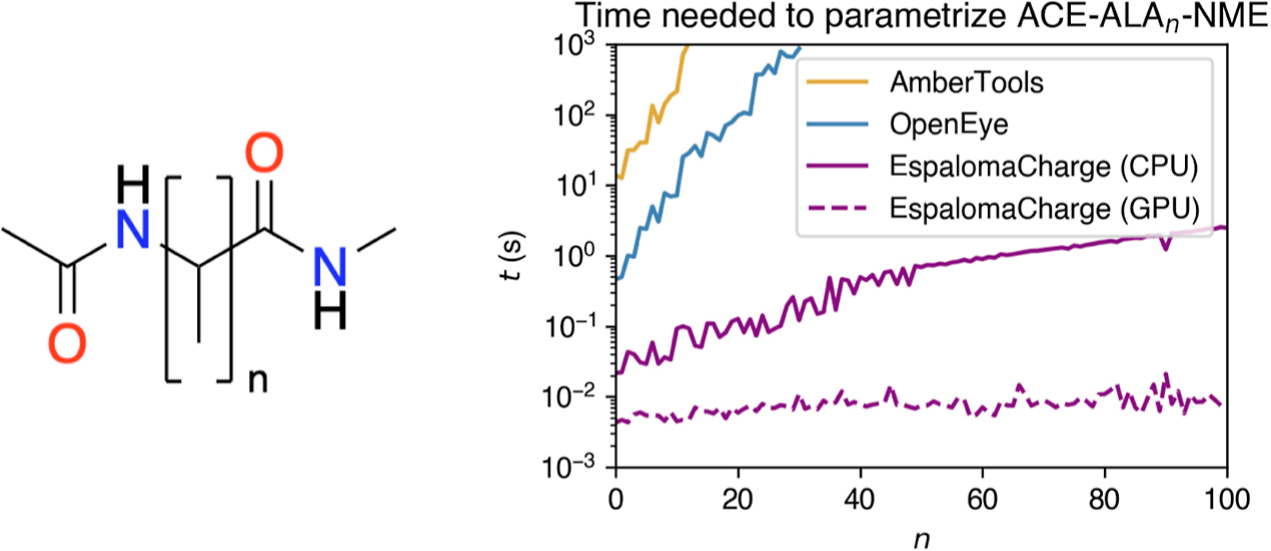
EspalomaCharge is fast, even for large systems. Wall time required to assign charges to ACE-ALA_*n*_-NME peptides with different toolkits is shown on a log plot, illustrating that EspalomaCharge on the CPU or GPU is orders of magnitude faster than semiempirical-based charging methods for larger molecules or biopolymers and is practical even for assigning charges to proteins of practical size. Fluctuation in traces is due to the stochasticity in timing trials.

We closely examine the dependence of parametrization time on the size of the (biopolymer) system in Figure 3, where we choose the peptide system ACE-ALA*n*-NME while varying *n* = 1, ..., 100. The parameterization wall time for AmberTools and OpenEye rapidly increases w.r.t. the size of the system (the theoretical runtime complexity for semiempirical methods are ) and exceeds 1000 s at *n* = 18 and *n* = 30, respectively. This scenario explains the infeasibility of employing AM1-BCC charges in parameterizing large systems. EspalomaCharge, on the other hand, has  complexity and is capable of parameterizing peptides of a few hundred residues within seconds. This process can be further accelerated by distributing calculations on the GPU hardware.
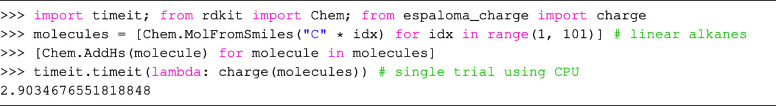


Batching many molecules into a single charging calculation can provide significant speed benefits when parameterizing large virtual libraries by making maximum use of hardware parallelism. EspalomaCharge provides a seamless way to achieve these speedups when providing a sequence of molecules, rather than single molecules at a time, as the input to the charge function in the API (Listing 5). In this case, the molecular graphs are batched with their adjacency matrix concatenated diagonally, processed by GNN and QEq models, and subsequently unbatched to yield the result. For instance, the wall time needed to parameterize all 100 ACE-ALA_*n*_-NME molecules from *n* = 1, ..., 100 depicted in Figure 3 at once, in batch mode, is 7.11 s with CPU—only marginally longer than the time required to parameterize the largest molecule in the data set, indicating that hardware resources are barely being saturated at this point.

### Error from Experiment in Explicit Solvent Hydration Free Energies Is Not Statistically Significantly Different between EspalomaCharge, AmberTools, and OpenEye Implemnetations of AM1-BCC

**Figure 4:**
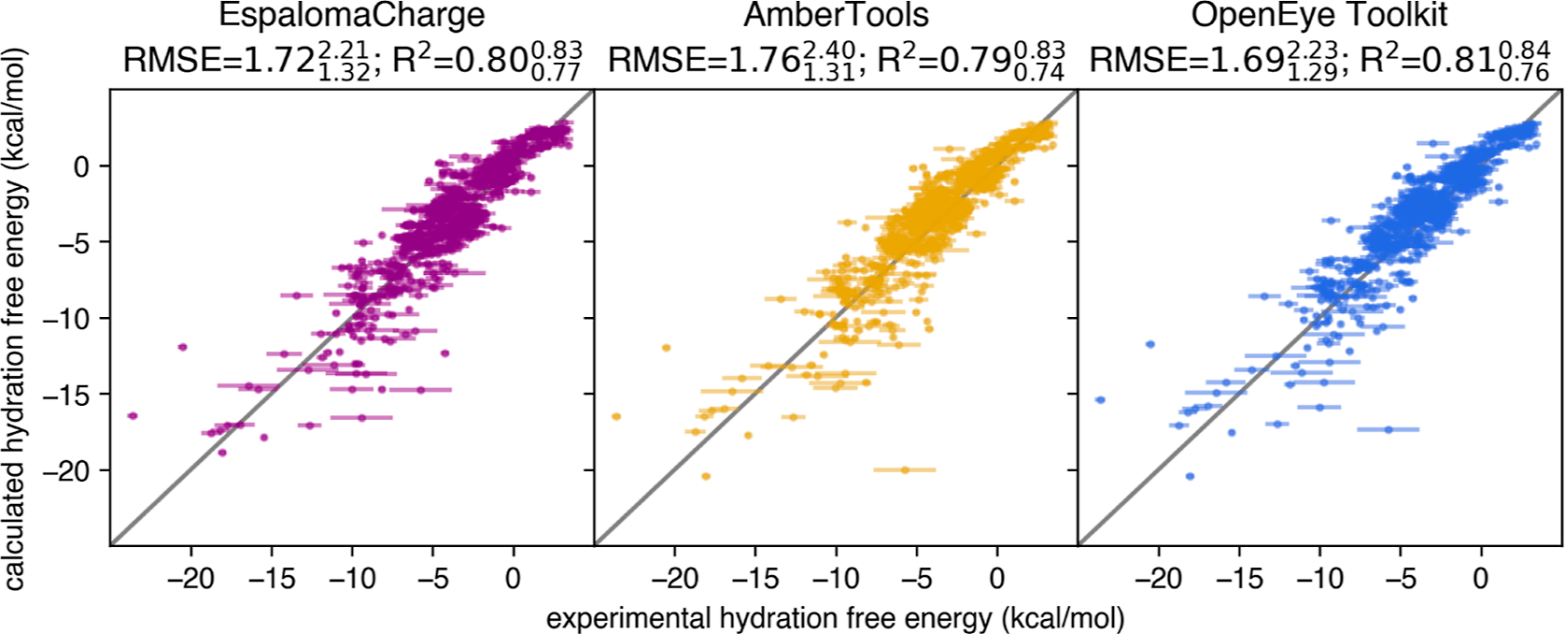
EspalomaCharge introduces little error to explicit hydation free energy prediction. Calculated-vs-experimental explicit solvent hydration free energies computed with AM1-BCC charges provided by EspalomaCharge, AmberTools, and the OpenEye Toolkit, respectively. Simulations used the GAFF 2.11 small molecule force field^28^ and TIP3P water^18^ with particle mesh Ewald electrostatics (see Detailed Methods section in Supporting Information). Annotated are RMSE and *R*^2^ score there between and bootstrapped 95% confidence interval. See also Appendix Figure S3 for comparison among computed hydration free energies.

While the charge deviations between EspalomaCharge and other toolkit implementations of AM1-BCC are comparable to the deviation between toolkits, it is unclear how the magnitude of these charge deviations translates into deviations of observable condensed-phase properties (such as free energies) from the experiment. To assess this, we carried out explicit solvent hydration free energy calculations, which serve as an excellent gauge of the impact of parameter perturbations,^24^ as the result is heavily dependent upon the small-molecule charges. We use each set of charges in calculating the hydration free energies for the molecules in FreeSolv^7^ (see Detailed Methods section in Supporting Information), a standard curated data set of experimental hydration free energies. In Figure 4, we compare the computed explicit solvent hydration free energies with experimental measurements and quantify the impact of the charge model on both deviation statistics (RMSE) and correlation statistics (*R*^2^) with the experiment. We note that EspalomaCharge provides statistically indistinguishable performance compared to AmberTools^5^ and the OpenEye toolkit on both metrics, RMSE and *R*^2^. This encouraging result suggests that any discrepancy introduced by EspalomaCharge is unlikely to significantly alter the qualitative behavior of MD simulations in terms of ensemble averages or free energies.

## Discussion

### EspalomaCharge Assigns High-Quality Conformation-Independent AM1-BCC Charges Using a Modern Machine Learning Infrastructure That Supports Accelerated Hardware

Composing the Espaloma graph neural networks framework^31,32^ for producing continuous, vectorial representations of the chemical environment of individual atoms with a conformation-independent QEq scheme^10^ for assigning partial atomic charges that satisfy total molecular charge constraints, EspalomaCharge provides a robust approach for assigning conformer-agnostic AM1-BCC charges to biomolecular systems. Because EspalomaCharge is built on PyTorch,^25^ a fast, modern, Python-based machine learning framework, it supports multiple optimized compute backends, including both CPUs and GPUs. Unlike AM1-BCC implementations based on traditional semiempirical quantum chemical codes, EspalomaCharge has  runtime complexity with respect to the number of atoms *N* (Figure 3) and introduces only small discrepancies to high-quality AM1-BCC reference implementations comparable to the discrepancies among popular AM1-BCC implementations (Table 1).

### Ability to Assign Topology-Driven Conformation-Independent Self-Consistent Charges to Small Molecules and Biopolymers Prepares the Community for Next-Generation Unified Force Fields

EspalomaCharge, thanks to its  runtime complexity, can assign charges to biopolymers with hundreds of residues—including proteins with exotic post-translational modifications or covalent ligands, nucleic acids, or complex conjugates of multiple kinds—within seconds. For the first time, rather than using multiple distinct methodologies to parametrize various components in a system (e.g., RESP-derived charges for amino acids and AM1-BCC charges for noncovalent ligands), it is feasible to simultaneously and self-consistently parametrize small molecules and biopolymers (and more complex covalent modifications of biopolymers) with a high-quality self-consistent scheme. This would be compatible with the next generation of unified force fields for small molecules and biopolymers, namely, Wang et al.^31^ Note that, although EspalmoaCharge can be employed to fit any atomic charges, in this paper, we only consider charge assignment schemes that are geometry-agnostic.

### EspalomaCharge Provides a Simple API and CLI for Facile Integration into Popular Workflows

EspalomaCharge is a pip-installable (Listing 1) open software package (see the Detailed Methods section in Supporting Information), making it easy to integrate into existing workflows with minimal complexity. Assigning charges to molecules using the EspalomaCharge Python API is simple and straightforward (Listing 2). A GPU can be used automatically, and entire libraries can be rapidly parameterized in batch mode (Listing 5). EspalomaCharge provides both a Python API and a convenient CLI, allowing EspalomaCharge to be effortlessly integrated into popular MM and MD workflows such as the OpenForceField toolkit (Listing 3) and Amber (Listing 4).

### One-Hot Embedding Cannot Generalize to Rare or Unseen Elements

One-hot element encoding is used in the architecture, making the model unable to perceive elemental similarities. This would compromise per-node performance for rare elements and prevent the model from being applied on unseen elements. Possible ways to mitigate this limitation include encoding the elemental physical properties as the node input.

### Future Expansions of the Training Set Could Further Mitigate Errors

As shown in Figure 2, the generalization error is heavily dependent on the data abundance within the relevant stratification of the training set—bins containing more training data show higher accuracy. Future work could aim to systematically identify underrepresented regions of chemical space and expand training data sets to reduce error for uncommon chemistries and exotic charge states, either with larger static training sets or using active learning techniques.

### Multiobjective Fitting Could Enhance Generalizability

Though EspalomaCharge produces an accurate surrogate for AM1-BCC charges, these small errors in charges can translate to larger deviations in ESP (see Supporting Information Figure S2). Since the function mapping charges (together with conformations) to ESPs are simple and differentiable, one can easily incorporate ESP as a target in the training process, using ESPs derived either from reference charges or (as in the original RESP^2^) to quantum chemical ESPs. A multiobjective strategy that includes multiple targets (such as charges and ESPs), potentially with additional charge regularization terms (as in RESP^2^), could result in more generalizable models with lower ESP discrepancies. Furthermore, similar observables can be incorporated into the training process to improve the utility of the model in modeling of real condensed-phase systems. For instance, condensed-phase properties, such as densities or dielectric constants, other quantum chemical properties, or even experimentally measured binding free energies.

## Supporting Information Available

Detailed methods—from code implementation, data set curation, to model training—used to produce the results in this paper (PDF)

## Notes

JDC is a current member of the Scientific Advisory Board of OpenEye Scientific Software, Redesign Science, Ventus Therapeutics, and Interline Therapeutics and has equal interest in Redesign Science and Interline Therapeutics. The Chodera laboratory receives or has received funding from multiple sources, including the National Institutes of Health, the National Science Foundation, the Parker Institute for Cancer Immunotherapy, Relay Therapeutics, Entasis Therapeutics, Silicon Therapeutics, EMD Serono (Merck KGaA), AstraZeneca, Vir Biotechnology, Bayer, XtalPi, Interline Therapeutics, the Molecular Sciences Software Institute, the Starr Cancer Consortium, the Open Force Field Consortium, Cycle for Survival, a Louis V. Gerstner Young Investigator Award, and the Sloan Kettering Institute. A complete funding history for the Chodera lab can be found at http://choderalab.org/funding. YW has limited financial interest in Flagship Pioneering, Inc. and its subsidiaries.

## Footnotes

a ELF10 denotes that the ELF (“electrostatically least-interacting functional groups”) conformer selection process was used to generate 10 diverse conformations from the lowest energy 2% of conformers. Electrostatic energies are assessed by computing the sum of all Coulomb interactions in vacuum using the absolute values of MMFF charges assigned to each atom.^13^ AM1-BCC charges are generated for each conformer and then averaged.
