# Supplementary material for: EspalomaCharge: Machine Learning-Enabled Ultrafast Partial Charge Assignment
Source: J Phys Chem A. 2024 May 8;128(20):4160–7. doi: 10.1021/acs.jpca.4c01287 (PMC11129294; doi:10.1021/acs.jpca.4c01287)
Supplement: Supplementary file 1 — jp4c01287_si_001.pdf [file jp4c01287_si_001.pdf]

Supporting Information: EspalomaCharge: Machine learning-enabled ultra-fast partial charge assignment

Yuanqing Wang* [1, 2], **Iván Pulido** [1], **Kenichiro Takaba** [1, 3], **Benjamin Kaminow** [1, 4], **Jenke Scheen** [1], **Lily Wang** [1, 5], **John D. Chodera*** [1]

[1]Computational and Systems Biology Program, Sloan Kettering Institute, Memorial Sloan Kettering Cancer Center, New York, NY 10065; [2]Simons Center for Computational Chemistry and Center for Data Science, New York University, New York, NY 10004; [3]Pharmaceutical Research Center, Advanced Drug Discovery, Asahi Kasei Pharma Corporation, Shizuoka 410-2321, Japan; [4]Tri-Institutional PhD Program in Computational Biology and Medicine, Weill Cornell Medical College, Cornell University, New York, NY 10065; [5]Open Molecular Sciences Foundation, Davis, CA 95618

***For correspondence:**
yuanqing.wang@choderalab.org (YW); john.chodera@choderalab.org (JDC)

Detailed methods

Code availability.

The Python code used to produce the results discussed in this paper is distributed open source under MIT license https://github.com/choderalab/espaloma_charge. Core dependencies include PyTorch 1.12.1 [22], Deep Graph Library 0.6.0 [29], and the Open Force Field Toolkit 0.11.2 [20].

Training dataset curation.

The SPICE [5] dataset is used as the training and in-distribution validation and test set for the model due to its thorough coverage of chemical space relevant to biomolecular simulations. It consists of druglike small molecules selected from PubChem, short peptides, and fragments of biomolecules and biopolymers, and covers 15 elements (H, Li, C, N, O, F, Na, Mg, P, S, Cl, K, Ca, Br, I). Protonation and tautomeric states have been enumerated for each molecule using the OpenEye toolkit. After random shuffling (over the chemical space, protomeric and tautomeric states are kept in the same partition), 80% of the dataset is used for training, 10% used for validation (and model selection via early stopping), and 10% for reporting out-of-sample test performance.

Out-of-distribution test dataset selection.

To test the generalizability of EspalomaCharge, we select a series of out-of-distribution test datasets on which the discrepancy between charge methods are assessed.

- **FDA approved** dataset[1] contains FDA approved small molecules, filtered by size and element composition.
- **ZINC250K** dataset is a popular machine learning dataset first published in Gómez-Bombarelli et al. [11], which randomly subsamples the original ZINC dataset [14].
- **FreeSolv** dataset [14] contains small molecules whose hydration free energies have been experimentally measured.

[1]Source: https://github.com/openforcefield/qca-dataset-submission/tree/master/submissions/2019-09-08-fda-optimization-dataset-1

Neural network architecture and training

Following the protocol specified in Wang et al. [30], we use GraphSAGE [13] as the GNN backbone and optimize the learning rate ($1e-2$ to $1e-5$), batch size (16 to 512), and neural network width (16 to 512) and depth (2 to 6) via grid search on the validation set. The input features of the atoms include the one-hot encoded element, as well as the hybridization, aromaticity, and (various sized-) ring membership, assigned using RDKit. Note that the formal charges are not included as part of the features to avoid the time-consuming enumeration of resonance structure as in Gilson et al. [9]. The hyperparameter search resulted in an optimal learning rate of 10^{-3} and L2 regularization with rate 10^{-4} with Adam optimizer [16] and batch size of 512; the neural networks are 4 layers and 128-unit wide. All models were trained for 5000 epochs, and the model with optimal performance on the validation set was selected for characterization here.

Electrostatic potential (ESP) errors

To calculate deviations between electrostatic potentials (ESP) on a surface, we first generated conformers using the OpenFF Toolkit 0.11.2. Conformer generation followed the Electrostatically Least-interacting Functional groups (ELF) approach. Initially, a maximum of 500 conformers was generated using RDKit with an RMS threshold of 0.05 Å. A *cis* conformation was enforced for carboxylic acid groups by rotating the protons in *trans* carboxylic acids 180° around the C-O bond. The electrostatic energy of each conformer was calculated using MMFF94 charges [12]. The 98% conformers with the highest electrostatic energy were discarded. From the remaining 2% conformers, we greedily selected up to 10 conformers that were most distinct from each other by RMS. Each conformer geometry was distinct by at least a heavy-atom RMS of 0.05 Å from each other.

For each conformer, we used OpenFF Recharge 0.4.0 to generate standard Merz-Singh-Kollman grids [25] around the molecule at a density of 1 point per Å^2. We then calculate the root mean squared error (RMSE) between ESPs generated by each set of partial charges on the conformer grid. To compare the overall effect of different partial charges on the ESP, we average the RMSE between ESPs for each conformer.

Induced solvent potential from Poisson-Boltzmann model (ZAP)

As a fast measure of how small differences in partial charges might impact interaction free energies, we computed the induced solvent potential on each atom using a fast Poisson-Boltzmann implicit solvation model implemented in OpenEye ZAP [10]. The induced solvent potential reflects the potential induced by the polarization of the solvent, and was computed following recommended standard usage [https://docs.eyesopen.com/toolkits/python/zaptk/thewayofzap.html].

Hydration free energies in explicit solvent (ΔG_{hyd})

To compute hydration free energies for the FreeSolv dataset [20] to quantify the impact of small differences in charges on experimentally-measurable free energies, we used a modified version of the protocol described in [21]. Neutral molecules were solvated with TIP3P water [15] in rectangular boxes with 14Å of padding, and assigned GAFF-2.11 parameters [27, 28] using openmmforcefields [3]. Hydration free energy calculations were computed by performing replica-exchange alchemical free energy calculations using a two-stage alchemical protocol in which charges were annihilated by linear scaling and Lennard-Jones interactions, and then subsequently annihilated using the Buetler softcore potential [1, 23]. Simulations employed particle mesh Ewald (PME) [7] to treat long-range electrostatics and used mixed precision to ensure accuracy in energies and integration. Integration was performed with the BAOAB Langevin integrator [17–19] using hydrogen masses of 3.8 amu to enable 4 fs timesteps to be taken while introducing minimal configuration space sampling error [8]. Calculations were carried out in gas phase at 298 K and in solvent at 1 atm using OpenMM 8 [6] and openmmtools 0.21.5 [2], and free energies were estimated with the multistate Bennett acceptance ration (MBAR) [24] after automatic equilibration detection [4] and decorrelation. Simulations were run for 1 ns/replica in each phase. Code for reproducing these calculations can be found in https://github.com/choderalab/espaloma_charge/tree/main/scripts/hydration-free-energies.

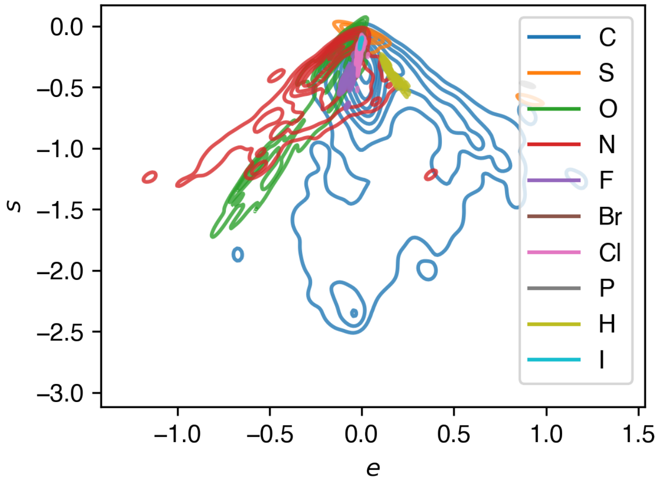

Figure S 1. EspalomaCharge provides interpretable intermediate representations. Kernel density estimate (KDE) plot of intermediate atomic electronegativity (e) and hardness (s) parameters used by the charge equilibration stage to generate charges, stratified by element. While physical instances of these parameters are limited to being positive, in this model they are unconstrained in sign.

References

[1] Beutler, T. C., Mark, A. E., van Schaik, R. C., Gerber, P. R., and Van Gunsteren, W. F. (1994). Avoiding singularities and numerical instabilities in free energy calculations based on molecular simulations. *Chemical physics letters*, 222(6):529–539.

[2] Chodera, J., Rizzi, A., Naden, L., Beauchamp, K., Grinaway, P., Fass, J., Wade, A., Rustenburg, B., Pulido, I., Ross, G. A., et al. (2022). choderalab/openmmtools: 0.21.5.

[3] Chodera, J., Wiewiora, R., Stern, C., and peastman (2020). openmm/openmm-forcefields: Fix GAFF AM1-BCC charging bug for some molecules.

[4] Chodera, J. D. (2016). A simple method for automated equilibration detection in molecular simulations. *Journal of chemical theory and computation*, 12(4):1799–1805.

[5] Eastman, P., Behara, P. K., Dotson, D. L., Galvelis, R., Herr, J. E., Horton, J. T., Mao, Y., Chodera, J. D., Pritchard, B. P., Wang, Y., et al. (2022). Spice, a dataset of drug-like molecules and peptides for training machine learning potentials.

[6] Eastman, P., Swails, J., Chodera, J. D., McGibbon, R. T., Zhao, Y., Beauchamp, K. A., Wang, L.-P., Simmonett, A. C., Harrigan, M. P., Stern, C. D., et al. (2017). Openmm 7: Rapid development of high performance algorithms for molecular dynamics. *PLoS computational biology*, 13(7):e1005659.

[7] Essmann, U., Perera, L., Berkowitz, M. L., Darden, T., Lee, H., and Pedersen, L. G. (1995). A smooth particle mesh ewald method. *The Journal of chemical physics*, 103(19):8577–8593.

[8] Fass, J., Sivak, D. A., Crooks, G. E., Beauchamp, K. A., Leimkuhler, B., and Chodera, J. D. (2018). Quantifying configuration-sampling error in langevin simulations of complex molecular systems. *Entropy*, 20(5):318.

[9] Gilson, M. K., Gilson, H. S. R., and Potter, M. J. (2003). Fast assignment of accurate partial atomic charges: An electronegativity equalization method that accounts for alternate resonance forms. *Journal of Chemical Information and Computer Sciences*, 43(6):1982–1997. PMID: 14632449.

[10] Grant, J. A., Pickup, B. T., and Nicholls, A. (2001). A smooth permittivity function for poisson–boltzmann solvation methods. *Journal of computational chemistry*, 22(6):608–640.

[11] Gómez-Bombarelli, R., Wei, J. N., Duvenaud, D., Hernández-Lobato, J. M., Sánchez-Lengeling, B., Sheberla, D., Aguilera-Iparraguirre, J., Hirzel, T. D., Adams, R. P., and Aspuru-Guzik, A. (2018). Automatic chemical design using a data-driven continuous representation of molecules. *ACS Central Science*, 4(2):268–276. PMID: 29532027.

[12] Halgren, T. A. (1996). Merck molecular force field. ii. mmff94 van der waals and electrostatic parameters for intermolecular interactions. *Journal of Computational Chemistry*, 17(5-6):520–552.

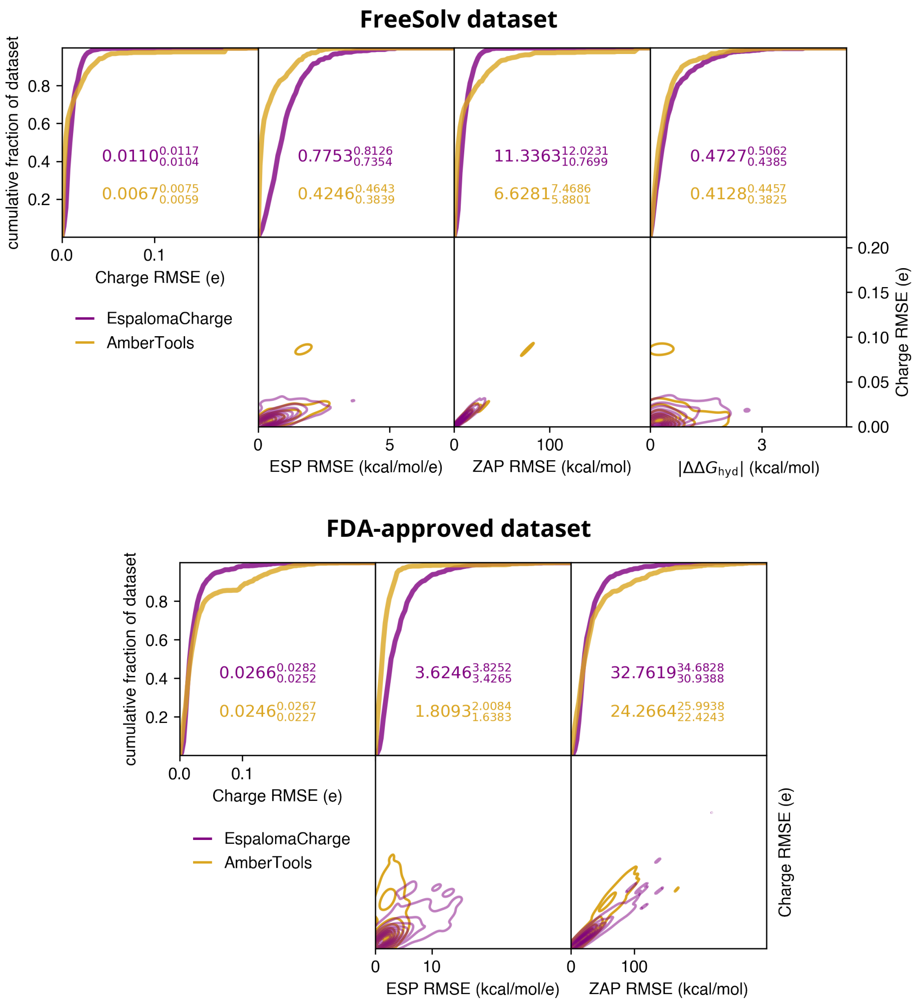

Figure S 2. Comparison of discrepancies the EspalomaCharge and AmberTools sqm produce in computing various charge-dependent properties, with high-quality OpenEye AM1-BCC ELF10 conformation-independent charges taken here as ground truth. The top row of each panel shows cumulative distribution functions (CDFs) of the deviations for each method, along with average (and 95% bootstrapped confidence intervals) for EsplomaCharge (grapefruit) and AmberTools (amber). The bottom row of each panel shows the joint probability density functions (PDFs) of the deviations for each method in various properties along with the charge RMSE. Here, **Charge RMSE (e)** denotes the root-mean squared (RMS) deviation of atomic charges for the molecule from OpenEye reference charges; **ESP RMSE (kcal/mol/e)** denotes the RMS deviation of electrostatic potential on surface shells from OpenEye reference charges; **ZAP RMSE (kcal/mol)** denotes the RMS deviation in the induced solvent potential computed via the OpenEye ZAP fast Poisson-Boltzmann implicit solvent model solver [10] between the query charge model and the OpenEye reference charges; $\Delta\Delta G_{\mathrm{hyd}}$ (kcal/mol) denotes the error in hydration free energies between the query charge model and the OpenEye reference charges. *Top panel:* The FreeSolv dataset [20] consists of 641 neutral small molecules with experimentally characterized hydration free energies. *Bottom panel:* A subset of 1615 FDA-approved inhibitors (retrieved from ZINC [26], originally sourced from DrugBank [31]) with elements compatible with EspalomaCharge.

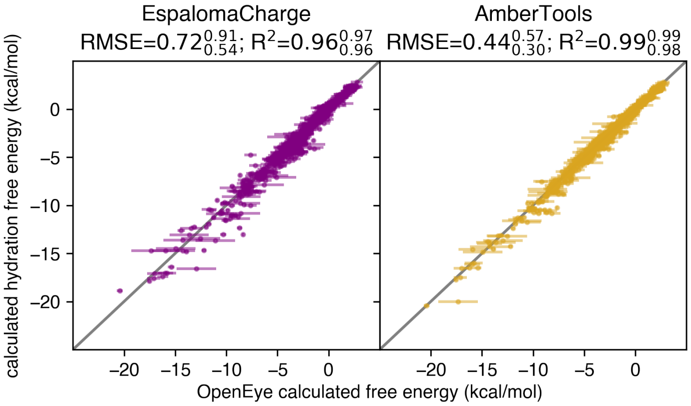

Figure S 3. EspalomaCharge introduces little error to explicit hydration free energy prediction. Hydration free energy with EspalomaCharge- and AmberTools-calculated partial charges plotted against that generated with OpenEye-computed charge.

---

[13] Hamilton, W., Ying, Z., and Leskovec, J. (2017). Inductive representation learning on large graphs. In *Advances in neural information processing systems*, pages 1024–1034.

[14] Irwin, J. J. and Shoichet, B. K. (2005). ZINC–a free database of commercially available compounds for virtual screening. *J Chem Inf Model*, 45(1):177–182.

[15] Jorgensen, W. L., Chandrasekhar, J., Madura, J. D., Impey, R. W., and Klein, M. L. (1983). Comparison of simple potential functions for simulating liquid water. *The Journal of chemical physics*, 79(2):926–935.

[16] Kingma, D. P. and Ba, J. (2015). Adam: A method for stochastic optimization. In Bengio, Y. and LeCun, Y., editors, *3rd International Conference on Learning Representations, ICLR 2015, San Diego, CA, USA, May 7-9, 2015, Conference Track Proceedings*.

[17] Leimkuhler, B. and Matthews, C. (2013a). Rational construction of stochastic numerical methods for molecular sampling. *Applied Mathematics Research eXpress*, 2013(1):34–56.

[18] Leimkuhler, B. and Matthews, C. (2013b). Robust and efficient configurational molecular sampling via langevin dynamics. *The Journal of chemical physics*, 138(17):05B601_1.

[19] Leimkuhler, B. and Matthews, C. (2016). Efficient molecular dynamics using geodesic integration and solvent–solute splitting. *Proceedings of the Royal Society A: Mathematical, Physical and Engineering Sciences*, 472(2189):20160138.

[20] Mobley, D. L., Bannan, C. C., Rizzi, A., Bayly, C. I., Chodera, J. D., Lim, V. T., Lim, N. M., Beauchamp, K. A., Shirts, M. R., Gilson, M. K., et al. (2018). Open force field consortium: Escaping atom types using direct chemical perception with smirnoff v0. 1. *BioRxiv*, page 286542.

[21] Mobley, D. L., Dumont, É., Chodera, J. D., and Dill, K. A. (2007). Comparison of charge models for fixed-charge force fields: small-molecule hydration free energies in explicit solvent. *The Journal of Physical Chemistry B*, 111(9):2242–2254.

[22] Paszke, A., Gross, S., Chintala, S., Chanan, G., Yang, E., DeVito, Z., Lin, Z., Desmaison, A., Antiga, L., and Lerer, A. (2017). Automatic differentiation in pytorch.

[23] Pham, T. T. and Shirts, M. R. (2012). Optimal pairwise and non-pairwise alchemical pathways for free energy calculations of molecular transformation in solution phase. *The Journal of chemical physics*, 136(12):124120.

[24] Shirts, M. R. and Chodera, J. D. (2008). Statistically optimal analysis of samples from multiple equilibrium states. *The Journal of chemical physics*, 129(12):124105.

[25] Singh, U. C. and Kollman, P. A. (1984). An approach to computing electrostatic charges for molecules. *Journal of Computational Chemistry*, 5(2):129–145.

[26] Sterling, T. and Irwin, J. J. (2015). Zinc 15–ligand discovery for everyone. *Journal of chemical information and modeling*, 55(11):2324–2337.

[27] Wang, J., Wang, W., Kollman, P. A., and Case, D. A. (2006). Automatic atom type and bond type perception in molecular mechanical calculations. *Journal of molecular graphics and modelling*, 25(2):247–260.

[28] Wang, J., Wolf, R. M., Caldwell, J. W., Kollman, P. A., and Case, D. A. (2004). Development and testing of a general amber force field. *Journal of computational chemistry*, 25(9):1157–1174.

[29] Wang, M., Zheng, D., Ye, Z., Gan, Q., Li, M., Song, X., Zhou, J., Ma, C., Yu, L., Gai, Y., et al. (2019). Deep graph library: A graph-centric, highly-performant package for graph neural networks. *arXiv preprint arXiv:1909.01315*.

[30] Wang, Y., Fass, J., Kaminow, B., Herr, J. E., Rufa, D., Zhang, I., Pulido, I., Henry, M., Bruce Macdonald, H. E., Takaba, K., and Chodera, J. D. (2022). End-to-end differentiable construction of molecular mechanics force fields. *Chem. Sci.*, 13:12016–12033.

[31] Wishart, D. S., Feunang, Y. D., Guo, A. C., Lo, E. J., Marcu, A., Grant, J. R., Sajed, T., Johnson, D., Li, C., Sayeeda, Z., et al. (2018). Drugbank 5.0: a major update to the drugbank database for 2018. *Nucleic acids research*, 46(D1):D1074–D1082.
